# Rapid detection of immunoglobulin heavy chain gene rearrangement by PCR and melting curve analysis using combined FR2 and FR3 primers

**DOI:** 10.1186/s13000-015-0370-5

**Published:** 2015-08-09

**Authors:** Danfei Xu, Zhuo Yang, Donghong Zhang, Wei Wu, Ye Guo, Qian Chen, Dongsheng Xu, Wei Cui

**Affiliations:** Department of Clinical Laboratory, Peking Union Medical College Hospital, Peking Union Medical College, Chinese Academy of Medical Sciences, Beijing, 100730 China; Hematopathology Program, CBL Path, Inc., Rye Brook, NY 10753 USA

## Abstract

**Background:**

Immunoglobulin heavy chain (IgH) gene rearrangement test is a standard tool in diagnosing B-cell lymphoma. The BIOMED-2 multiplex PCR protocol has become the most commonly used laboratory method for detecting clonal IgH gene rearrangement. However, post-PCR procedure requires manual transfer of PCR product for analysis and is time-consuming. A novel strategy using LightCycler to continuously monitor fluorescence during melting curve analysis (MCA) can overcome these shortcomings. The previous studies published on this method were all restricted to FR3 primers of BIOMED-2.

**Methods:**

Real-time PCR and subsequent MCA were performed on 71 clinical DNA samples from formalin-fixed, paraffin-embedded tissues, including 40 with B-cell non-Hodgkin lymphomas and 31 with reactive lymphoid hyperplasia. We optimized the current method using FR3 primers and applied FR2 primers for the first time into MCA to detect IgH gene rearrangement. Polyacrylamide gel electrophoresis and capillary gel electrophoresis were also performed on all lymphoma samples with the identical FR2 primers.

**Results:**

MCA of combined FR2 and FR3 primer sets yielded the sensitivity and the specificity equal to 70 % (28/40) and 100 % (31/31), respectively. Addition of FR2 primers increased the sensitivity by 12.5 % (5/40) comparing to FR3 primers alone. MCA was slightly more sensitive than polyacrylamide gel electrophoresis and comparable to capillary gel electrophoresis to detect clonal IgH gene rearrangement.

**Conclusions:**

Combined PCR and DNA melting curve analysis in a closed system can reduce cross-contamination risk. This method can test 96 samples simultaneously within 90 min and therefore, it is high-throughput and faster. PCR-MCA in the LightCycler system has potential for evaluating monoclonal IgH gene rearrangement in a clinical environment.

## Background

Non-Hodgkin B-cell lymphoma accounts for a significant proportion of malignant lymphomas [1–3]. Despite well-established diagnostic criteria, such as morphology and immunophenotyping, the clonality testing by immunoglobulin heavy chain (IgH) gene rearrangement is required in approximately 10 % of cases [4]. The IgH gene contains many different variable (V), diversity (D), and joining (J) segments, which are subject to randomly rearranged processes during early B-cell development, creating a unique hallmark for each B-cell [5]. V segments contain three framework regions (FRs) and two complementarity determining regions (CDRs). Unlike the FRs that are similar among various V segments, the CDRs are highly variable even within the same V family [6]. Since B-cell malignancies contain identically rearranged IgH genes, PCR priming at FRs is able to detect monoclonal B-cell population in the form of single band on gel or sharp peak on fragment analyser [7–9]. Of many DNA-based PCR tests, the protocol developed as a result of BIOMED-2 collaborative study [6] using three sets of primers named FR1, FR2 and FR3 has become the most commonly used laboratory method.

Current DNA-based PCR methods for detecting monoclonal IgH gene rearrangement (IgH-R) include polyacrylamide gel electrophoresis (PAGE), capillary gel electrophoresis (CE) or denaturing gradient gel electrophoresis [7–10]. These methods consist primarily of two separate steps: PCR amplification and post-PCR amplicon analysis. Xu et al. [11] developed a novel PCR method for detecting clonal IgH-R that continuously monitored fluorescence of the specific double-stranded DNA binding dye SYBR green I during melting curve analysis (MCA) on LightCycler. This method that used the FR3 primer set of BIOMED-2 was faster and reliable, and combined PCR and DNA MCA in a closed system reduced post-PCR cross-contamination risk. In this report, we reinvestigate PCR with FR3 primers and MCA strategy using the newest modal of LightCycler (Roche LightCycler 480 II). We have improved the analytical sensitivity using FR3 primers. In addition, we include FR2 primers for the first time into the protocol, and demonstrate that PCR in LightCycler system with melting curve analysis using combined FR2 and FR3 primers can increase the detection sensitivity, and is a reliable and reproducible method to diagnose the monoclonal IgH gene rearrangements.

## Methods

### Patient samples

Total 71 formalin-fixed paraffin-embedded (FFPE) tissue samples were collected during 2012 to 2014 at Peking Union Medical College Hospital. Fourty were from patients with B-cell non-Hodgkin lymphoma (B-NHL), and 31 were from patients with reactive lymphoid hyperplasia. All cases were definitively diagnosed by the pathologists and hematologists according to the World Health Organization system for the classification of hematolymphoid neoplasms. A mixture of 17 DNA samples from reactive lymphoid hyperplasia and Ramos DNA were used as negative and positive controls, respectively.

### DNA extraction

A microscopic examination was first made on a 5-μm thick H&E section to confirm the presence of suspected tumor cells of at least 80 %. Then, five 5-μm thick sections were consecutively cut and placed in a 1.5 ml tube. DNA was extracted according to the instructions from the TIANamp FFPE DNA kit (Tiangen, Beijing, China). The concentration of DNA was measured by NanoDrop (Thermo Fisher Scientific, Shanghai, China). The quality of each DNA sample was evaluated by amplifying a 333-bp fragment of the immunoglobulin M (Cμ).

### PCR primers

Two sets of primers referring to BIOMED-2 design [6] were synthesized by Invitrogen Biotechnology including IgH variable framework region 2 (FR2) and framework region 3 (FR3) plus joining region (JH). The primers for Cμ were designed by Primer-BLAST that amplified a constant region of immunoglobulin μ chain. The sequences were as followings:

- Cμ-F: 5’-GGCAAGGCCAAGAACTGTCT-3’;
- Cμ-R: 5’-TACATGGGCATCTCAAGGGG-3’.

### LightCycler multiplex PCR and melting curve analysis (MCA)

DNA from each sample was amplified in the LightCycler 480 II (Roche Diagnostic, Shanghai, China) by three primer sets, FR2, FR3 and Cμ, in three independent tubes. The reaction mixture of each well contained SYBR® Premix Ex Taq™ II master (Takara BIO, Dalian, China) 10 μl, 10 μmol/l of each primer 0.4 μl, genomic DNA 100 ng, and dH_2_O PCR grade with a final volume of 20 μl [12]. The PCR conditions were the same for all primer sets as followings: 95 °C 15 s, 62 °C 15 s and 72 °C 20 s. After 40 amplification cycles, MCA was performed. Briefly, PCR products were denatured at 95 °C for 1 min, annealed at 62 °C for 15 s and quickly raised to 75 °C, the temperature was then raised slowly from 75 °C to 93 °C at a transition rate of 0.05 °C/s during continuous fluorescence monitoring at 521 nm. Results were analysed by the Tm Calling software (Roche Diagnostic).

### Gel detection – PAGE (Heteroduplex analysis)

The IgH Tube B Master Mixture (InVivoScribe Technologies) that contains identical FR2 primer set was used to amplify the IgH gene variable region in a thermal cycler (BIO-RAD, Berkeley, CA, USA). 100 ng of genomic DNA was added up to a final reaction volume of 50 μl. After an initial “hot start” using AmpliTaq Gold (Applied Biosystems) at 95 °C for 7 min, the PCR cycle parameters were set as followings: denaturing at 95 °C for 45 s, followed by annealing at 60 °C for 45 s, and extending at 72 °C for 90 s. This program was repeated for 35 cycles. A final extension was performed at 72 °C for 10 min. For PAGE analysis, 10 μl of PCR products were denatured at 94 °C for 5 min and quickly chilled at 4 °C for 60 min. Each sample was mixed with 2 μl of 5x loading buffer before added on a 6 % non-denaturing acrylamide gel. The gel was run at room temperature for 50 min at 120 volts, stained with 0.5 % of GelRed (Fanbo Biochemicals, Beijing, China) and then visualized using UV illuminator (BIO-RAD, USA). The expected size range of the amplicons varied from 250 to 295 bp.

### Capillary gel electrophoresis (CE)

Premix Ex Taq master (Takara BIO, Dalian, China) and hexachloro (HEX) fluorescence labelled FR2 primers were used. PCR reaction system and condition were the same as those mentioned in the section of LightCycler multiplex PCR and MCA. The amplified fragments were analysed by ABI 3130 Genetic Analyzer (Applied Biosystems). Briefly, 1 μl of the PCR product was transferred to 10 μl of Hi-Di formamide with ROX size standards (InVivoScribe technology). Samples were denatured at 95 °C for 5 min, immediately chilled on ice for 2 min and analysed by the Peak Scanner software (Life Technology). The expected sizes varied from 250 to 295 bp.

## Results

### Establishment of LightCycler multiplex PCR and MCA

**Fig. 1:**
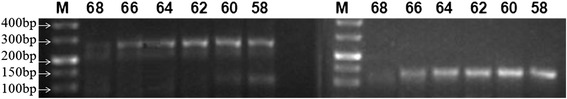
Annealing temperature gradient PCR on Ramos cell line. FR2 (left) and FR3 (right) primers were used to amplify Ramos cell line DNA with gradient annealing temperature from 58 °C to 68 °C to explore the optimal annealing temperature for both multiplex-PCR sets

**Fig. 2:**
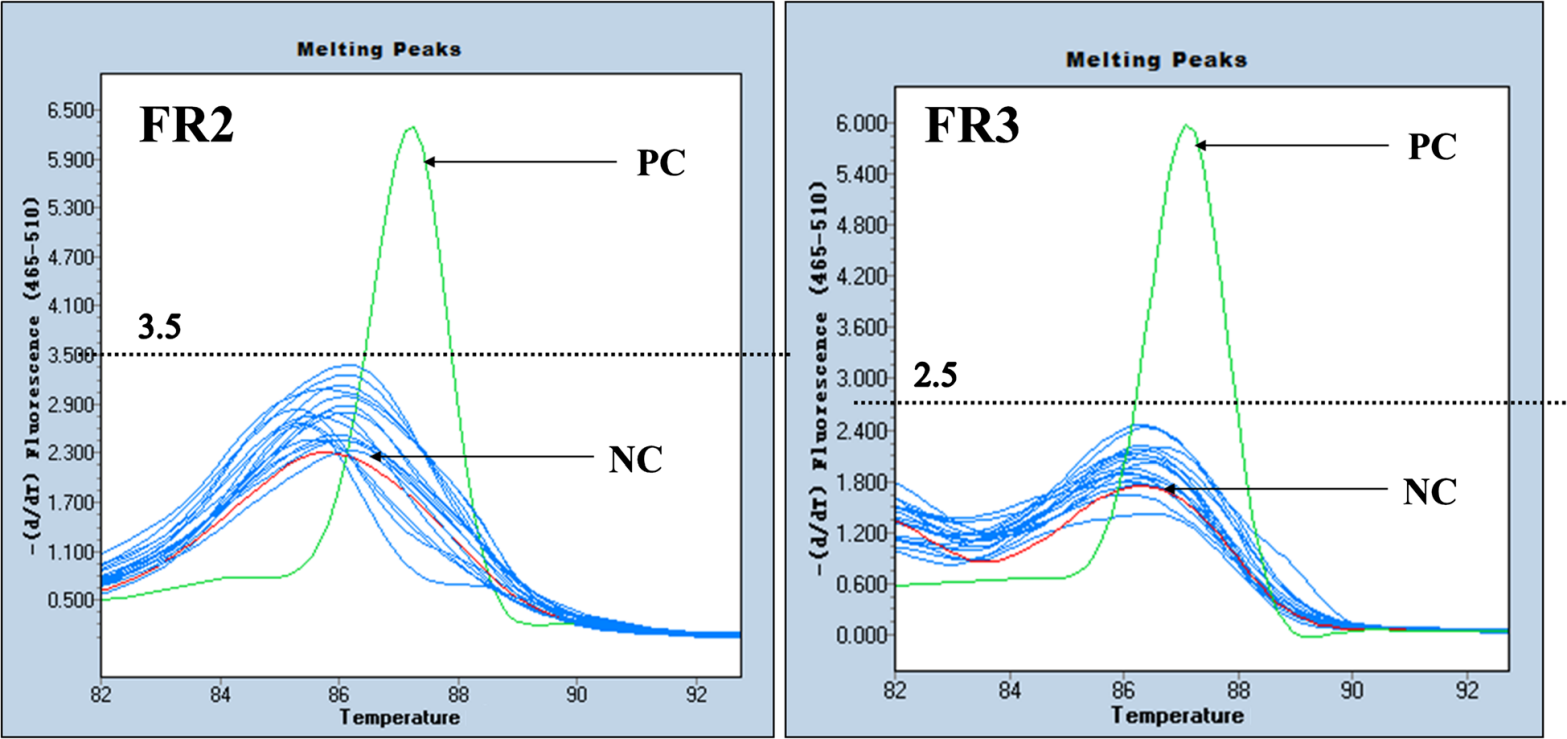
Melting curve analysis of 17 lymphoid hyperplasia samples. NC: negative control (red line); PC: positive control (green line); X axis = temperature (°C); Y axis = −dF/dT, where F = fluorescence and T = temperature

An annealing temperature gradient PCR on Ramos (Human B-cell non-Hodgkin lymphoma cell line) DNA from 58 °C to 68 °C was run to determine the optimal annealing temperature for multiplex PCR. According to the agarose gel electrophoresis results, the annealing temperature of 62 °C was suitable for both FR2 and FR3 primers (Fig. 1). Based on the previous studies [9, 10], 40 cycles of LightCycler PCR were sufficient for DNA amplification. Therefore, 62 °C and 40 cycles were used in this study. The 17 reactive lymphoid hyperplasia samples were individually tested by MCA. The result showed that all reactive samples as well as the negative control had flat, wide, irregular peaks with -dF/dT values less than 3.5 for FR2 or less than 2.5 for FR3. On the other hand, the positive control yielded a single sharp peak with a distinct Tm (Fig. 2). Therefore, the criteria for diagnosing a monoclonal IgH gene rearrangement were established as having a single sharp peak with the -dF/dT value equal to or greater than 3.5 for FR2 and 2.5 for FR3, respectively.

### Capability of distinguishing lymphoma in clinical FFPE samples

A total of 71 FFPE samples were analysed by PCR-MCA. All 31 lymphoid hyperplasia samples had –dF/dT values <3.5 for FR2 and <2.5 for FR3, resulting in the specificity of 100 %. Among 40 lymphoma samples, 28 (70.0 %) samples were positive for monoclonal IgH gene rearrangement (Table 1) including 5 with FR2 rearranged, 5 with FR3 rearranged, and 18 with both FR2 and FR3 rearranged. Therefore, inclusion of FR2 primers increased the sensitivity by 12.5 % (5/40) comparing to use the FR3 primers alone. The Tms of 23 FR2 monoclonal clinical samples plus Ramos cell line ranged from 84 °C to 88 °C with the mean of 86.4 °C and the Tms of 23 FR3 monoclonal clinical samples plus Ramos cell line ranged from 83 °C to 89 °C with the mean of 86.1 °C (Table 1).

**Table 1:** Summary of clinical lymphoma samples analysed by MCA, PAGE and CE

Patient	Age/sex	Diagnosis	Tissue	MCA-FR3(Tm°C)	MCA-FR2(Tm°C)	PAGE-FR2	CE-FR2(nt)
1	39/F	SLL	Inguinal LN	P(84.78)	P(86.34)	P	P(295)
2	53/F	MCL	Intestine	P(86.17)	P(86.83)	P	P(251)
3	54/F	CLL/SLL	Lung	P(85.44)	N	N	N
4	61/F	MCL	Neck Skin	P(87.95)	P(87.68)	P	P(254)
5	54/F	CLL/SLL	Tonsil	P(86.94)	P(87.18)	P	P(253)
6	61/F	MZL	Spleen	P(84.93)	P(87.03)	P	P(285)
7	51/F	DLBCL	Lung	P(84.89)	P(85.20)	P	P(286)
8	47/F	B-NHL-UC	Brain	P(85.72)	N	N	N
9	63/M	MZL	Neck Skin	P(86.17)	P(86.96)	P	P(277)
10	49/M	MZL	Parotid Gland	P(88.54)	N	N	N
11	66/F	CLL/SLL	Inguinal LN	P(86.80)	P(86.48)	P	P(260)
12	77/F	B-NHL-UC	Eyelid	P(86.52)	P(85.60)	P	P(254)
13	75/F	B-NHL-UC	Tongue	P(84.83)	P(85.50)	P	P(280)
14	27/M	MZL	Parotid Gland	P(85.80)	P(86.64)	P	P(273)
15	43/M	MZL	Lung	P(84.88)	P(86.69)	P	P(265)
16	56/M	DLBCL	Spleen	N	N	N	N
17	65/F	B-NHL-UC	Spleen	P(85.67)	N	N	N
18	68/M	B-NHL-UC	Inguinal LN	N	P(87.14)	P	P(265)
19	51/F	FL	Neck Skin	P(87.77)	P(87.64)	P	P(259)
20	40/F	DLBCL	Ovary	N	N	N	N
21	74/M	MCL	Tonsil	P(84.21)	P(85.14)	P	P(277)
22	28/F	FL	Axillary LN	N	N	N	N
23	50/M	DLBCL	Nasopharyngeal LN	N	N	N	N
24	61/F	MZL	Spleen	P(83.10)	P(87.16)	P	P(272)
25	75/F	DLBCL	Pelvic Tumor	P(86.52)	P(86.14)	UD	P(272)
26	44/M	B-NHL-UC	Axillary LN	P(87.45)	P(87.35)	UD	P(272)
27	73/F	MZL	Mandibular Gland	N	P(85.08)	P	P(259)
28	68/F	DLBCL	Colon	N	N	N	N
29	63/F	DLBCL	Axillary LN	P(86.96)	N	N	P(254/263)
30	62/M	FL	Colon	N	N	N	N
31	70/F	MZL	Stomach	N	N	N	N
32	72/M	DLBCL	Pectoral LN	N	N	N	N
33	77/F	DLBCL	Cheek Skin	N	N	N	N
34	62/M	DLBCL	Spleen	N	P(86.25)	P	P(259)
35	79/F	DLBCL	Tonsil	N	N	N	N
36	58/M	DLBCL	Lung	N	P(85.18)	P	P(242)
37	65/F	MZL	Thyroid	N	N	N	N
38	38/M	MZL	Conjunctiva	N	P(84.45)	P	P(262)
39	62/M	DLBCL	Neck Skin	P(86.82)	P(86.30)	P	P(248)
40	62/F	DLBCL	Stomach	N	N	N	N

The Tms of clonal samples tested by MCA and segment length from CE are listed in the parentheses

*CLL/SLL* chronic lymphocytic leukemia/small lymphocytic lymphoma, *DLBCL* diffuse large B-cell lymphoma, *FL* follicular lymphoma, *MCL* mantle cell lymphoma, *MZL* marginal zone B-cell lymphoma, *B-NHL-UC* unclassified B-NHL, *LN* Lymph node, *P* positive, *N* negative, *UD* undetermined

### Comparison of MCA with PAGE and CE

**Fig. 3:**
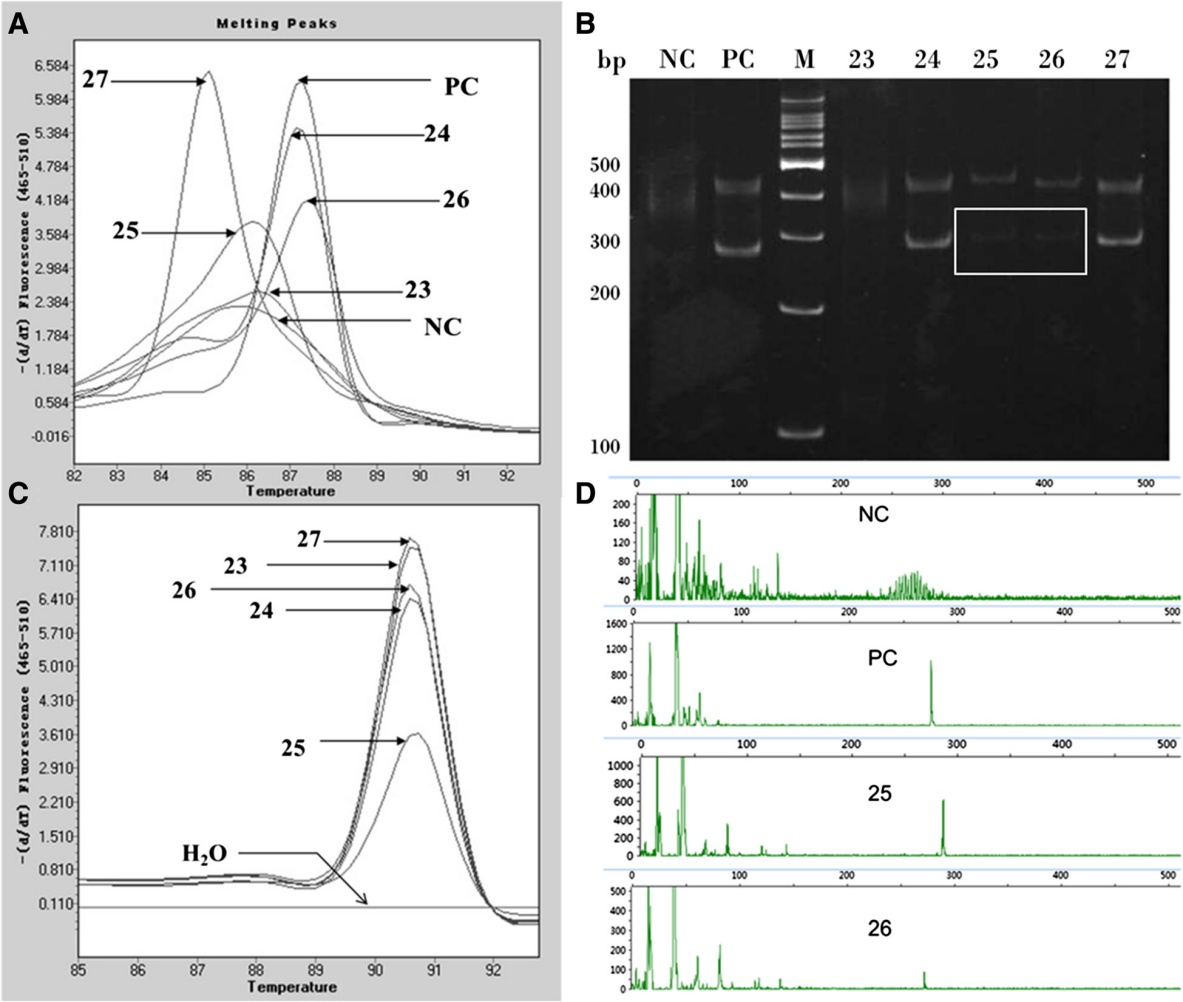
Comparison of PAGE, MCA and CE. **a** MCA of 5 lymphoma samples with FR2 primers. Sample 23 was negative, 24–27 were positive. **b** PAGE of the same 5 lymphoma samples. A very faint band can hardly be seen in sample 25# and 26# (in box) that is interpreted as undetermined and need to be further confirmed. **c** Evaluation of DNA quality. All samples including 25# and 26# have a sharp peak at identical Tm, which indicated the presence of lymphoid tissue with integrated DNA. **d** CE of the sample 25# and 26#. Both samples had a single peak at expected sizes

Conventional BIOMED-2 protocol (PAGE) versus MCA for detecting monoclonal IgH gene rearrangement was compared in 40 clinical B-NHL samples. All negative cases by MCA were also negative by PAGE. For 23 FR2-positive cases detected by MCA, PAGE clearly detected 21 positive cases. The cases 25# and 26# showed very faint band respectively on PAGE at expected range (Fig. 3b). We interpreted the results as undetermined that need to be further confirmed. Melting curve analysis of internal control Cμ showed that these two samples had a specific peak (Fig. 3c). The fragments analysis by Peak Scanner showed the PCR products of the two samples have a monoclonal peak with designated sizes (Fig. 3d), consistent with MCA results. Of all 40 clinical B-NHL samples, CE detected 24 positive cases with FR2 primers. One showed biallelic rearrangement in CE, but neither MCA nor PAGE demonstrated a positive result (Table 1). To further study the capability of MCA in distinguishing biallelic or bi-cloanl rearrangements with FR2 primers, equal amounts of two different clonal DNA samples were mixed together, PCR-amplified with FR2 primer set and analysed using melting curve analysis (see below).

### Minimal detection of MCA

**Fig. 4:**
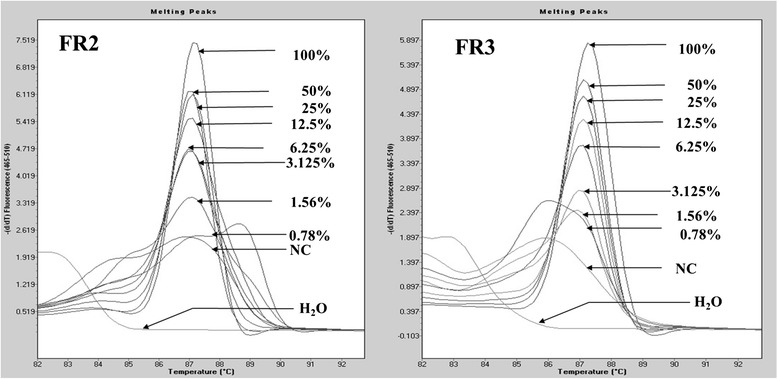
Minimal detection of MCA. Ramos cell line DNA was serially diluted with negative control at various percentages (50 %, 25 %, 12.5 %, 6.25 %, 3.125 %, 1.56 % and 0.78 %). After PCR, a clonal peak at 3.125 % can still been detected using both FR2 and FR3 primer sets

To determine the minimal detection of IgH gene rearrangement with MCA, serial dilutions of Ramos cell line with the negative control at various percentages of 50 %, 25 %, 12.5 %, 6.25 %, 3.125 %, 1.56 % and 0.78 % were prepared. After 40 cycles of amplification, we could still detect a single sharp peak at 3.125 % with –dF/dT values greater than 3.5 for FR2 and 2.5 for FR3, respectively on melting curve analysis (Fig. 4).

### Reproducibility of MCA

Three consecutive melting curve analyses of DNA from Ramos cell line and FR2 positive clinical DNA samples yielded identical -dF/dT versus T curves. The average Tm SD of all clonal DNA samples was 0.048. On the other hand, three consecutive melting curve analyses of DNA samples from reactive lymphoid hyperplasia produced different -dF/dT versus T curves each time with an average Tm SD of 0.428. Therefore, we recommend performing three consecutive MCAs after DNA amplification to exclude the pseudo-clonal high peak rarely observed with reactive lymphoid hyperplasia reported previously [13].

### Resolution of MCA using FR2 primers

To test whether MCA could distinguish biallelic of bi-clonal rearrangements, equal amounts of clonal DNA samples with two different Tms were mixed, PCR-amplified with FR2 primers and analysed using melting curve analysis (Table 2). We were not able to separate two clonal B-cell populations with △Tm less than 1.05 (Experiments 1 and 2). However, we were able to distinguish two clonal B-cell populations with △Tm equal to or greater than 2.03 °C (Table 2, experiments 3–5).

**Table 2:** MCA of two different clonal DNA samples using FR2 primers

Experiment NO.	Original			After mixing	
	Tm1	Tm2	△Tm	Tml	Tm2
1	84.45	85.18	0.73	84.45	—
2	84.45	85.50	1.05	84.62	—
3	84.45	88.48	2.03	84.41	86.56
4	84.45	87.35	2.90	84.11	86.99
5	84.45	87.68	3.23	84.23	87.50

△Tm: Difference of melting temperatures of two original samples individually detected by MCA

## Discussion

The melting temperature (Tm) of double stranded DNA reflects fundamental physical properties that depend on length, sequence, G: C content and Watson-Crick base pairing [14–16]. Xu et al. [11] originally demonstrated that melting curve analysis based on internal melting domain found in CDRs can distinguish monoclonal from polyclonal IgH gene rearrangements. The PCR products from monoclonal B-cell population will present a single, sharp and symmetric peak at certain Tm on MCA, while those amplicons from polyclonal B-cell population will show flat, wide and irregular peaks. Xu et al. [11] showed that PCR-MCA yielded 100 % of sensitivity (24/24) compared to PAGE and 58.3 % of sensitivity (7/12) compared to southern blot analysis. Since then, several studies using PCR-MCA strategy to detect monoclonal IgH rearrangement have been reported [17–19]. Dobbs et al. [17] used an iCycler instrument and achieved 88.9 % sensitivity, compared to PAGE. Retamales et al. [19] reported a 95.2 % of sensitivity compared to PAGE, and concluded that PCR-MCA could distinguish reliably between monoclonal versus polyclonal IgH rearrangement in gastric lymphoid infiltrates. Compared to capillary gel electrophoresis (CE) and PAGE, Kummalue et al. [18] achieved 88.5 % of sensitivity in 26 cases of B-cell non-Hodgkin lymphoma. Steiff et al. [20] found the similar sensitivity of MCA versus CE to detect clonal IgH rearrangement in 130 cases of gastric biopsy specimen. Furthermore, Uemura et al. [21] reported that PCR-MCA yielded 89.2 % of sensitivity compared to southern blot analysis (42/45), while PAGE showed only 77.8 % of sensitivity (35/45). All these reported studies targeting at CDR3 with FR3 primers showed no false positive results, resulting in consistent 100 % of specificity.

In this study, we reinvestigate PCR with FR3 primers and MCA strategy using the newest modal of LightCycler. We have improved the analytical sensitivity from originally reported 12.5 % [11] to current 3.125 %. We believed that the newer modal of LightCycler has a more precise temperature control system than the previous one. In addition, the reagents we used in this study are the optimal premix master that has minimized the PCR-suppression effect of SYBR Green I. All these may have contributed to a higher analytic sensitivity.

We also incorporate FR2 primers for the first time into the protocol. Combination of FR2 and FR3 primer sets enables us to detect 28 cases of (70.0 % sensitivity) monoclonal IgH-R in 40 B-NHL FFPE samples, which detects 12.5 % more cases than using FR3 primers alone. PCR-MCA with FR2 primers appears slightly more sensitive than PAGE (23 vs 21) and shows comparable sensitivity to CE (95.8 %). The diagnostic sensitivity in this study was different from that reported by McClure et al. (60.8 %) [22], which detected 28 clonal IgH-R cases in 46 B-cell neoplasm FFPE samples using capillary gel electrophoresis with BIOMED-2 primers. We believe that the different lymphoma types and PCR reagents used in two studies may account for the discrepancy in detection rate. The Tm range of the 23 FR2 positive cases was from 84 °C to 88 °C and the Tm range of FR3 cases we obtained was close to that reported by Xu et al. [11]. None of the 31 reactive lymphoid hyperplasia samples shows clonal peak, giving the specificity once again of 100 %.

## Conclusions

In summary, to our knowledge, this is the first study combining FR2 and FR3 primers to determine IgH-R by melting curve analysis in LightCycler system, which is based on a fundamental DNA characteristic. Precise temperature control produces accurate and reproducible Tms. The results are reliable and promising with analytic as well as clinical sensitivities comparable with those of capillary gel electrophoresis. However, PCR-MCA in LightCycler has several advantages. This method can test 96 samples simultaneously within 90 min. Therefore, it is high-throughput and faster. In addition, combined PCR and DNA melting curve analysis in a closed system reduces cross-contamination risk. Furthermore, it is more accessible than fragment analyzer for most hospitals in developing countries. Primers sets, such as FR1 of IGH or IGK may also be developed using MCA in the future to further improve overall sensitivity. We believe that PCR-MCA in the Light Cycler system has potential for evaluating monoclonal IgH-R in a clinical environment.

## Abbreviations

B-NHL: B-cell non-Hodgkin lymphoma
CDRs: Complementarity determining regions
CE: Capillary gel electrophoresis
Cμ: Immunoglobulin M
FFPE: Formalin-fixed paraffin-embedded
FR2: Framework region 2
FR3: Framework region 3
Ig: Immunoglobulin
IgH: Immunoglobulin heavy chain
IgH-R: IgH rearrangement
MCA: Melting curve analysis
PAGE: Polyacrylamide gel electrophoresis
Tm: Melting temperature
